# Prevalence of bacillary layer detachment in diabetic macular edema and response to 3 anti-vascular endothelial growth factor treatment

**DOI:** 10.1097/MD.0000000000035576

**Published:** 2023-10-20

**Authors:** Yann-Guang Chen, Yun-Hsiang Chang, Hsin-Ching Shen, Shu-I. Pao, Yu-Chih Hou, I-Chia Liang

**Affiliations:** a Department of Ophthalmology, Tri-Service General Hospital, National Defense Medical Center, Taipei, Taiwan, R.O.C.; b Department of Ophthalmology, National Taiwan University Hospital Yunlin Branch, Douliu City, Yunlin, Taiwan, R.O.C.; c Department of Ophthalmology, National Taiwan University Hospital, Taipei, Taiwan, R.O.C.; d Department of Ophthalmology, Cathay General Hospital, Taipei, Taiwan, R.O.C.

**Keywords:** anti-vascular endothelial growth factor, bacillary layer detachment, diabetic macular edema

## Abstract

Spectral-domain optical coherence tomography is widely used in maculopathy, including diabetic macular edema (DME). Bacillary layer detachment (BALAD) is a novel optical coherence tomography finding, defined as the separation of the intraretinal layer between the inner segment myoids and ellipsoids. A total of 161 treatment-naïve eyes with centrally involved DME that underwent 3 monthly loading doses of anti-vascular endothelial growth factor (anti-VEGF) intravitreal injections were enrolled and analyzed retrospectively. BALAD was found in 6.2% of eyes with concurrent subretinal fluid (SRF). All eyes were divided into 3 groups: no either group had neither SRF or BALAD; the SRF only group had SRF but no BALAD; and the BALAD group had both SRF and BALAD. A significant increase in baseline central foveal thickness (CFT) in the BALAD group was observed (no either vs SRF only vs BALAD, baseline CFT: 387.6 ± 74.29 vs 440.6 ± 106.79 vs 642.0 ± 188.86; *P *< .01). Total resolution of BALAD was noted after anti-VEGF therapy, along with a significant decrease in CFT in all groups (CFT decrease: 82.4 ± 87.07 vs 187.6 ± 138.88 vs 252.1 ± 127.63; *P *< .01). Eyes with BALAD tended to have the worst baseline visual acuity (baseline logarithm of the minimum angle of resolution VA: 0.76 ± 0.353 vs 0.63 ± 0.303 vs 1.15 ± 0.300; *P* = .046) but showed the most improvement after treatment (logarithm of the minimum angle of resolution VA change: −0.14 ± 0.235 vs −0.22 ± 0.275 vs −0.27 ± 0.250; *P* = .079). After resolution of BALAD, all eyes in the BALAD group exhibited ellipsoid zone and/or interdigitation zone disruption corresponding to the BALAD area. BALAD is a novel optical coherence tomography finding associated with a spectrum of diseases including DME. With anti-VEGF therapy, total resolution of BALAD and a significant decrease in CFT can be obtained. However, ellipsoid zone/interdigitation zone disruption tended to develop.

## 1. Introduction

Diabetes mellitus has become one of the most prominent chronic illnesses in practically all countries because of lifestyle changes that encourage less physical exercise and a worrying rise in obesity. Compared to 2010, the number of patients with diabetes in underdeveloped nations would rise by 69%, while that in industrialized countries would rise by 20%.^[1]^

One of the leading factors in global blindness and moderate to severe vision impairment is diabetic retinopathy^[2,3]^ and it is the primary contributor to blindness among people of working age in developed countries.^[3]^ The most prevalent microvascular consequence of diabetes is also referred to as diabetic retinopathy,^[4]^ and can be found in some form in over 70% of patients with type 1 diabetes and over 50% of those with type 2 diabetes after 15 years of disease,^[5]^ with an increase in disease severity correlating with the duration of diabetes.^[5,6]^

The main risks of potential vision loss and blindness in diabetic patients are mainly caused by diabetic macular edema (DME) and proliferative diabetic retinopathy,^[4]^ with DME as a result of leakage of retinal vasculature in the non-proliferative phase and proliferative diabetic retinopathy stemming from angiogenesis and highly permeable capillaries in the proliferative phase.^[7]^

In recent years, improvements in diagnostic and therapeutic techniques have coped with large-scale clinical needs, and there are still unmet needs that must be addressed. Optical coherence tomography (OCT) is now widely acknowledged as a new reference standard for evaluating DME owing to the growing availability of OCT instruments, as well as their accuracy and capacity to provide information on retinal layer structure.^[8,9]^ It is used as both a diagnostic tool and a means of assessing treatment effects and disease progression.^[8,9]^ With the appearance of newer models, such as spectral domain OCT (SD-OCT) devices, further anomalies that might not have been picked up in the past can now be clearly visualized.^[10]^

Bacillary layer detachment (BALAD) was first described by Mehta et al^[11]^ in a case of toxoplasmosis chorioretinitis and pachychoroid disease, when an intraretinal layer separation occurring between the inner segment (IS) myoids and ellipsoids resulted in a distinctive dome-shaped or piriform-shaped cystic intraretinal space on SD-OCT.

BALAD has been reported in various studies over the years, encompassing several case reports, case series, and retrospective studies spanning a spectrum of diseases, including toxoplasmosis chorioretinitis,^[11]^ Vogt-Koyanagi-Harada disease,^[12]^ neovascular macular degeneration,^[13]^ acute idiopathic maculopathy,^[14]^ central serious chorioretinopathy,^[15]^ serpiginoid choroiditis,^[16]^ blunt eye trauma with choroidal rupture,^[17]^ posterior scleritis,^[18]^ intraocular tuberculosis,^[19]^ acute posterior multifocal placoid pigment epitheliopathy,^[20]^ fungal endophthalmitis,^[21]^ sympathetic ophthalmia,^[22]^ and also DME.^[23]^

In this retrospective study, we included treatment-naïve DME patients who received 3 monthly anti-vascular endothelial growth factor (anti-VEGF) injections and used SD-OCT to determine the prevalence and response to anti-VEGF treatment in eyes with DME either simultaneously presenting with or without BALAD.

## 2. Materials and methods

### 2.1. Ethical review

The study was conducted in accordance with the Declaration of Helsinki, and approved by the Institutional Review Board of Tri-Service General Hospital (protocol code #A202005165, date of approval:2020/12/08) and Cathay General Hospital (protocol code #P109090, date of approval:2021/02/16).

### 2.2. Participants

The medical records of patients over 18-year-old and with type 2 diabetes mellitus and DME at the Tri-Service General Hospital and Cathay General Hospital were reviewed from January 2016 to December 2020. Eyes with a diagnosis of central-involved DME on OCT and a central foveal thickness (CFT) more than or equal to 300um, completed 3 strict monthly loading doses of intravitreal injection of anti-VEGF agents with either ranibizumab 0.5 mg/0.05 mL or aflibercept 2.0 mg/0.05 mL, and fulfilled the following examination regimen, were included. Before anti-VEGF treatment, all patients underwent a blood test for hemoglobinA1c (HbA1c) and fluorescein angiography (FA) to identify the severity of diabetic retinopathy (non-proliferative or proliferative), and underwent a detailed ocular evaluation at baseline, which included a slit lamp examination, indirect ophthalmoscopy, color fundus photography, SD-OCT, and best-corrected visual acuity (BCVA) in logarithm of the minimum angle of resolution (logMAR) units. All patients were observed 2 weeks after each injection and received routine eye examinations, including logMAR BCVA measurements, fundus examinations with dilated pupils or color fundus photography, and OCTs, which were collected for analysis in this study. Both eyes were included if they met our inclusion criteria; however, baseline tests were performed independently if DME was diagnosed at different periods. BCVA and CFT on OCT were subsequently used to determine treatment effectiveness.

Eyes with BCVA worse than logMAR 1.3, cloudy media or extreme refractive errors affecting fundus observation and other retinal pathologies that can cause macular edema or affect visual function were excluded.

### 2.3. OCT characteristics and BALAD determination

Vertical and horizontal OCT images across the fovea were obtained using SD-OCT (Zeiss Cirrus 5000-HD-OCT, Carl Zeiss Meditec, Dublin, CA, or RTVue XR, Optovue, Fremont, CA). Characteristics on OCTs performed at baseline were assessed according to previous articles including (1) the presence or absence of subretinal fluid (SRF); (2) the presence or absence of vitreomacular interface disorders including epiretinal membrane or vitreomacular traction; (3) the presence or absence of intraretinal cysts (in the outer nuclear layer, or in the inner nuclear layer, or in both layers); (4) the presence or absence of hyper-reflective foci (HRF) and the quantity of HRF (few < 11, moderate 11–20, many > 20), (5) the presence or absence of ellipsoid zone (EZ) disruption; and (6) the presence or absence of disorganization of retinal inner layers (between ganglion cell-inner plexiform layer and inner nuclear layer, or between inner nuclear layer and outer plexiform layer, or both).^[24,25]^

The diagnosis of BALAD was made based on OCT images and was defined as an intraretinal space splitting apart the hyporeflective IS myoid zone, with the anterior border, or the “ceiling,” taking the form of a granular band, and the posterior border of the BALAD, or the “floor,” as a continuation of the EZ of adjacent retina though showing attenuation at the bottom of the split space.^[26]^

To corroborate the diagnosis of all pretreatment OCT characteristics, 2 retinal specialists independently inspected the images. After treatment, whether the macular edema (including the presence or absence of any intraretinal fluid [IRF] or SRF) existed or not were also evaluated by these 2 retinal specialists via interpreting the OCT images gathered 2 weeks after each intravitreal injection. The pre- and posttreatment OCTs were interpreted separately but under a blinded mechanism; the interpreting retinal specialists would not know the pretreatment condition while interpreting posttreatment OCTs. If both specialists made the same diagnosis for the same patient, the patient was allocated to their respective diagnosis. Nonetheless, if the 2 specialists disagreed regarding the patient’s diagnosis, a third retinal specialist was consulted to confirm the diagnosis, with the majority opinion serving as the final decision.

### 2.4. Statistical analysis

All statistical analyses were performed with MedCalc Software.

Baseline data included age, sex, HbA1c value, lens status (phakia or pseudophakia), severity of diabetic retinopathy (proliferative or non-proliferative), logMAR BCVA, and CFT, and were compared between all groups using either analysis of variance (continuous variables) or chi-square test (categorical variables). The manifestations of various OCT characteristics were compared among all groups using the chi-squared test.

Post hoc analysis with the Student–Newman–Keuls test was used to determine differences between paired groups.

There was no missing data need to be addressed.

*P*-value < 0.05 was considered statistically significant.

## 3. Results

A total of 161 eyes of 116 patients were included in our study. All eyes had different degrees of various types of IRF. BALAD were identified in 6.2% (10 of 161 eyes) of the study eyes. All eyes with BALAD demonstrated SRF. Subsequently, we divided all study eyes into 3 groups: no either group (DME with IRF but no SRF and no BALAD), SRF only group (DME with IRF and SRF, but no BALAD), and BALAD group (DME with IRF, SRF, and BALAD).

### 3.1. Baseline characteristics

As shown in Table 1, we classified the baseline and posttreatment anatomical parameters and measurements of 161 research eyes. Age was similar in all 3 groups, though the age of the BALAD group had a tendency to be younger as opposed to the other 2 groups (no either group vs SRF only group vs BALAD group, 65.1 ± 8.07 vs 61.3 ± 6.95 vs 57.5 ± 8.19; *P* = .154). Sex and HbA1c levels were similar in the eyes of all 3 groups (*P* = .588 and 0.371, respectively). Lens status was similar in all 3 groups, although no either group had a relatively higher ratio of pseudophakic eyes (phakia: pseudophakia, no either group vs SRF-only group vs BALAD group, 74:41 vs 29:7 vs 8:2; *P* = .137). The severity of diabetic retinopathy was also similar in all 3 groups (*P* = .674).

**Table 1:** Patient characteristics before and after 3 loading doses of anti-vascular endothelial growth factor.

Group	No either	SRF only	BALAD	*P*-value
	IRF *present*			
	SRF			
	*Absent*	*Present*		
		BALAD		
		Absent	Present	
No. of eyes	115	36	10	
Age (yrs)	65.1 ± 8.07 (38–90)	61.3 ± 6.95 (47–76)	57.5 ± 8.19 (40–76)	.154
Sex (M:F)	63:52	21:15	4:6	.588
HbA1c	7.84 ± 1.312 (4.8–13.6)	8.03 ± 1.332 (5.0–13.6)	7.63 ± 1.237 (5.8–13.6)	.371
Lens status (phakia: pseudophakia)	74:41	29:7	8:2	.137
DR (NPDR:PDR)	64:51	20:16	7:3	.674
BCVA (logMar)	0.76 ± 0.353 (0.0–1.3)	0.63 ± 0.303 (0.3–2.0)	1.15 ± 0.300 (0.3–1.3)	.046
BL-CFT (um)	387.6 ± 74.29 (305–850)	440.6 ± 106.79 (322–835)	642.0 ± 188.86 (359–793)	<.001
*OCT characteristics*				
Vitreomacular interface (normal:ERM:VMT)	77:6:32	25:3:8	8:0:2	.774
Intraretinal cyst (absent: present)	14:101	2:34	2:8	.360
Hyperreflective foci (<11:11–20:>20)	75:31:9	14:13:9	3:3:4	.002
EZ disruption (absent: present)	39:76	3:33	0:10	.002
DRIL (absent: present)	22:93	6:30	0:10	.307
*Post 3 IVI*				
logMAR VA improvement after 3 IVI (postIVI-baseline)	−0.14 ± 0.235 (−0.9–0.8)	−0.22 ± 0.275 (−0.8–0.3)	−0.27 ± 0.250 (−0.6–0)	.079
CFT decrease after 3 IVI (baseline-postIVI) (um)	82.4 ± 87.07 (−127–401)	187.6 ± 138.88 (−73–590)	252.1 ± 127.63 (78–475)	<.001

BALAD = bacillary layer detachment, BCVA = best corrected visual acuity, BL-CFT = baseline central foveal thickness, DR = diabetic retinopathy, DRIL = disorganization of retinal inner layer, ERM = epiretinal membrane, EZ = ellipsoid zone, HbA1c = hemoglobin A1c, IRF = intraretinal fluid, IVI = intravitreal injection, NPDR = non-proliferative DR, OCT = optical coherence tomography, PDR = proliferative DR, SRF = subretinal fluid, VMT = vitreomacular traction.

### 3.2. Baseline BCVA and CFT

The BALAD group tended to have poorer BCVA at baseline compared to the other 2 groups (no either group vs SRF-only group vs BALAD group, 0.76 ± 0.353 vs 0.63 ± 0.303 vs 1.15 ± 0.300; *P* = .046). The BALAD group had the thickest baseline CFT, followed by the SRF alone group, with the no either group having the thinnest baseline CFT (no either group vs SRF only group vs BALAD group, 387.6 ± 74.29 vs 440.6 ± 106.79 vs 642.0 ± 188.86; *P* < .01).

### 3.3. OCT characteristics

OCT characteristics revealed similar results for vitreomacular interface disorder, intraretinal cyst, and disorganization of retinal inner layers at baseline (*P* = .774, .699, and .724, respectively). HRF increased significantly in the SRF only and BALAD groups compared to the no either group (*P* < .01). The destruction of the EZ structure was more extensive in the SRF only and BALAD groups than in the no either group (*P* = .023).

### 3.4. BALAD presentation

**Figure 1.**
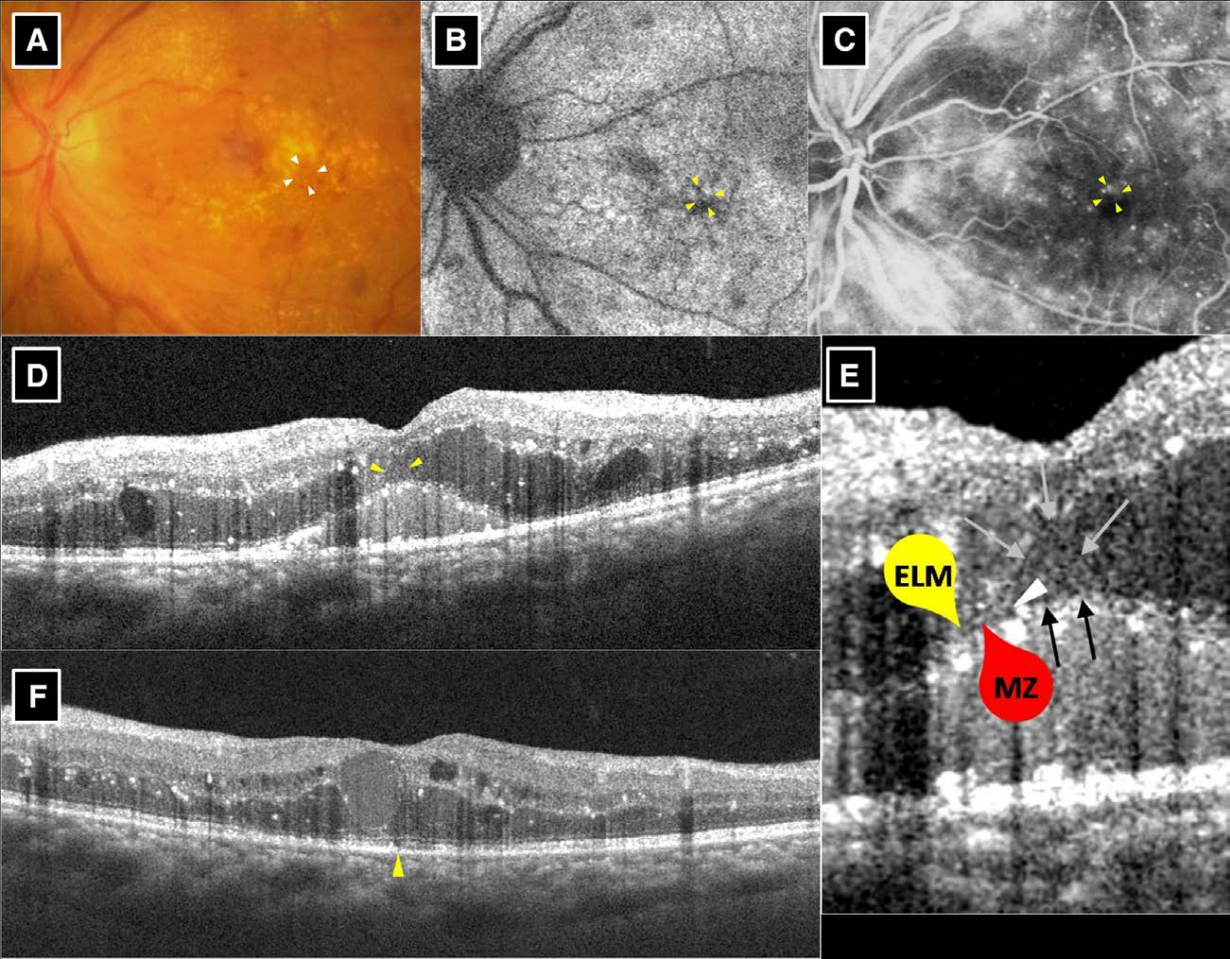
Multimodel imaging of eye 3 in bacillary layer detachment (BALAD) only group. Pretreatment (A–E). Posttreatment (F). (A) Color fundus photo. Typical findings of diabetic macular edema (DME) including microaneurysm, retinal hemorrhage, and hard exudate were noted. The border of BALAD represented as a yellowish ring (arrowheads). (B) Fundus autofluorescence. BALAD exhibited hypo-autofluorescence with a border of hyper-autofluorescence (arrowheads). (C) Fluorescein angiography. Typical findings of DME including microaneurysm and leakage were noted. The border of BALAD revealed as a ring of hyperfluorescence (arrowheads). (D) Spectral-domain optical coherence tomography (SD-OCT). Typical findings of DME including intraretinal fluid (IRF), cystic edema and subretinal fluid (SRF) were noted. Foveal BALAD was demonstrated (arrowheads). (E) Magnified view of D. The external limiting membrane (ELM) (yellow marker), myoid zone (MZ) (red marker), separation of myoid (white arrowhead), the roof (gray arrows) and floor (black arrows) of the BALAD were exhibited. The space between the roof and floor is the BALAD. E, SD-OCT after 3 anti-vascular endothelial growth factor treatments. Complete resolution of BALAD was noted with a small disruption of ellipsoid and interdigitation zone (arrowhead) left. SRF subsided thoroughly with decrease of IRF and cystic edema.

**Figure 2.**
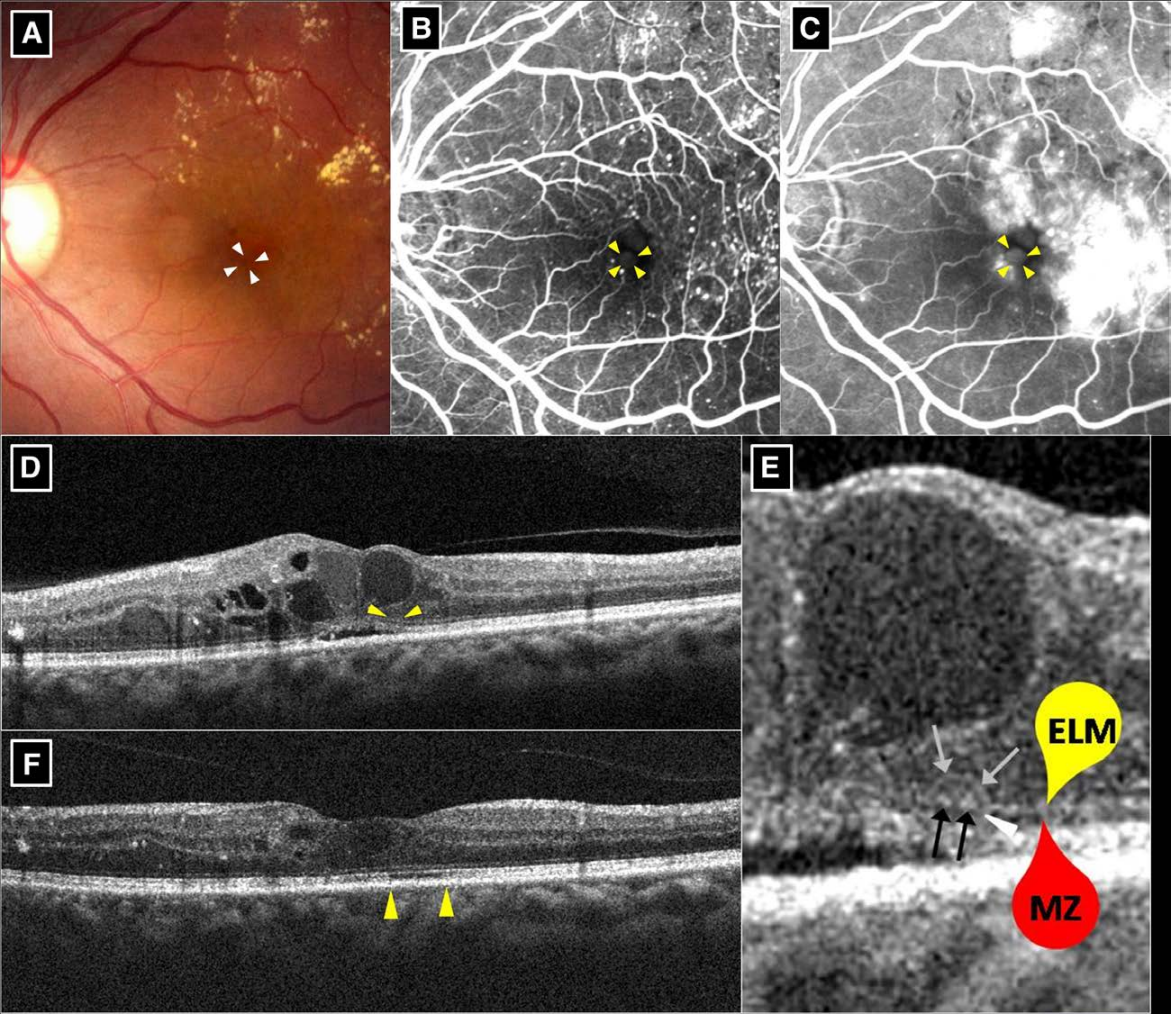
Multimodel imaging of eye 5 in bacillary layer detachment (BALAD) only group. Pretreatment (A–E). Posttreatment (F). (A) Color fundus photo. Hard exudate and microaneurysm representative of diabetic macular edema (DME) were noted. The border of BALAD represented as a yellowish ring (arrowheads). (B and C) Early and late phase of fluorescein angiography. Typical findings of DME including microaneurysm and leakage were noted. The BALAD revealed as a ring of hyperfluorescence with pooling of dye (arrowheads). (D) Spectral-domain optical coherence tomography (SD-OCT). Typical findings of DME including intraretinal fluid (IRF), cystic edema and subretinal fluid (SRF) were noted. Foveal BALAD was demonstrated (arrowheads). (E) Magnified view of D. The external limiting membrane (ELM) (yellow marker), myoid zone (MZ) (red marker), separation of myoid (white arrowhead), the roof (gray arrows) and floor (black arrows) of the BALAD were exhibited. The space between the roof and floor is the BALAD. (E) SD-OCT after 3 anti-VEGF treatments. Complete resolution of BALAD was noted with an attenuation of EZ and disruption of interdigitation zone (arrowheads) left. SRF subsided thoroughly with decrease of IRF and cystic edema.

Baseline details, posttreatment parameters, and measurements of the 10 eyes in the BALAD group are shown in Table 2. Multimodal images of the 2 representative cases of BALAD are shown in Figure 1 (eye 3) and Figure 2 (eye 5). The border of the BALAD presented as a yellowish ring on the color fundus photograph (Figs. 1A and 2A). A relatively hypo-autofluorescent lesion with a thin hyper-autofluorescent contour on a fundus autofluorescence photo was observed (Fig. 1B). The same contour was observed on FA (Figs. 1C and 2B), along with pooling of dye within the BALAD area (Fig. 2C). On SD-OCT, BALAD appeared as a space separating the myoid zone with hyperreflective particles contained therein. The anterior border of the BALAD, or the “ceiling,” was a granular band that almost fused with the overlying external limiting membrane (ELM). The posterior border of the BALAD, or the “floor,” was continuous with the EZ of the adjacent retina (Figs. 1D, F and 2D, F). After the resolution of BALAD following 3 intravitreal injections, all eyes in the BALAD group showed disruption of the EZ and/or interdigitation zone (IZ) in the region corresponding to BALAD (Figs. 1E and 2E). The SRF subsided completely in 9 eyes after 3 intravitreal injections, while minimal SRF left in the other one eye (eye 1), which had large amount of SRF before treatment. The IRF and cystic edema partially improved in all 10 eyes after 3 intravitreal injections.

**Table 2:** The baseline details and the posttreatment parameters and measurements of the 10 eyes in the BALAD group.

Eye	Baseline														Post 3 IVI				
	Age (yrs)	Gender	HbA1c (mg/dL)	Lens status	DR severity	Tx Hx	Anti-VEGF	BCVA (logMAR)	CFT (um)	VM interface disorder	Intraretinal cysts	Hyperreflective foci	EZ disruption	DRIL	BCVA (logMAR)	CFT (um)	BALAD resolution	SRF resolution	EZ/IZ disruption develop under BALAD
1	66	F	6.4	Phakia	PDR	PRP	Ranibizumab	1.3	793	No	Yes	11–20	Yes	Yes	0.7	456	Complete	Partial	Yes
2	63	F	7.7	Pseudophakia	NPDR	nil	Aflibercept	1.0	782	No	Yes	>20	Yes	Yes	0.5	307	Complete	Complete	Yes
3	51	F	7.1	Phakia	NPDR	nil	Aflibercept	1.3	602	No	Yes	>20	Yes	Yes	1.3	488	Complete	Complete	Yes
4	53	M	13.6	Phakia	NPDR	nil	Ranibizumab	0.7	569	ERM	Yes	<11	Yes	Yes	0.2	297	Complete	Complete	Yes
5	58	F	6.8	Pseudophakia	NPDR	nil	Ranibizumab	0.3	442	No	Yes	<11	Yes	Yes	0.2	281	Complete	Complete	Yes
6	40	M	7.3	Phakia	PDR	PRP	Ranibizumab	0.3	584	No	Yes	<11	Yes	Yes	0.2	228	Complete	Complete	Yes
7	56	M	6.6	Phakia	NPDR	nil	Aflibercept	1.3	641	No	Yes	>20	Yes	Yes	1.3	317	Complete	Complete	Yes
8	55	F	6.5	Phakia	NPDR	PRP	Aflibercept	1.3	359	No	Yes	11–20	Yes	Yes	1.3	230	Complete	Complete	Yes
9	76	F	5.8	Phakia	NPDR	nil	Aflibercept	0.7	588	No	Yes	>20	Yes	Yes	0.3	313	Complete	Complete	Yes
10	50	M	9.3	Phakia	PDR	PRP and PPV	Aflibercept	1.0	391	ERM	No	11–20	Yes	Yes	0.5	313	Complete	Complete	Yes

BCVA = best corrected visual acuity, CFT = central foveal thickness, DR = diabetic retinopathy, DRIL = disorganization of retinal inner layer, ERM = epiretinal membrane, EZ = ellipsoid zone, HbA1c = hemoglobin A1c, IVI = intravitreal injection, IZ = interdigitation zone, NPDR = non-proliferative DR, PDR = proliferative DR, PPV = pars plana vitrectomy, PRP = panretinal photocoagulation, Tx Hx = treatment history or diabetic retinopathy, VEGF = vascular endothelial growth factor, VM = vitreomacular.

### 3.5. BCVA and CFT change after 3 monthly loading doses of anti-VEGF injections

After 3 monthly intravitreal injections of anti-VEGF, the BALAD group showed the greatest improvement in BCVA while the SRF only group was slightly better than the no either group, although there was no significant difference between the 3 groups (logMAR VA change, no either group vs SRF only group vs BALAD group, −0.14 ± 0.235 vs −0.22 ± 0.275 vs −0.27 ± 0.250; *P* = .079). A significant decrease from baseline CFT was evident after 3 doses of intravitreal injections in the BALAD group and the SRF only group compared with the no either group (CFT decrease, no either group vs SRF only group vs BALAD group, 82.4 ± 87.07 vs 187.6 ± 138.88 vs 252.1 ± 127.63; *P *< .01).

## 4. Discussion

Our results showed that BALAD was found in 6.2% (10 of 161) of the eyes with DME. The BALAD noted in this study is shown on SD-OCT as a splitting space with highly reflective particles inside, which separated the IS myoid zone between 2 hyper-reflective borders, with the posterior border shown as the hyper-reflective EZ. The presence of BALAD is always associated with SRF, as observed in our study. A significant improvement in CFT after anti-VEGF treatment was also noted, along with a trend towards improvement in BCVA in eyes with BALAD. In a recent case report by Amini et al also revealed a 55-year-old female with diabetic retinopathy was treated with a single dose of anti-VEGF and resulted in increased visual acuity and complete remission of BALAD.^[23]^

On fundus autofluorescence photo, BALAD was hypo-autofluorescent. This observation was similar to that of previous studies, and it was hypothesized that this was due to masking of retinal pigment epithelium (RPE) autofluorescence caused by intra-BALAD exudation, RPE disruption, or both.^[26]^ Late-phase FA demonstrated dye pooling in the BALAD cavity; however, there was less dye pooling in our instances compared to prior reports.^[26]^This may be due to the less serous but more exudative components of the BALAD, the hyperfluorescent leaking of nearby microaneurysms, or both.

Solitary SRF, outer retinal tubulations (ORTs),^[27,28]^ and cystoid macular edema are OCT anomalies that may mimic bacillary separation. SRF is the deposition of fluid between the neurosensory retina and RPE, while BALAD is an outer retinal hyporeflective gap separating the IS myoid zone. Although ORTs are likewise defined by an outer retinal hyporeflective space,^[26,27,29]^ they are typically round or ovoid in shape, have a thick but smooth hyper-reflective border, and are more anterior than BALADs despite being located inside the outer retina. BALADs frequently contain scattered hyper-reflective particles, are piriform or dome-shaped, and have an outer border that is hyper-reflective and continuous with the EZ layer.^[27,29]^ In addition, ORTs are seldom foveal, whereas BALADS are commonly foveal or parafoveal.^[30]^ ORTs are non-exudative and resistant to anti-VEGF treatment, which is another key difference between bacillary detachments and ORTs.^[27]^ Cystoid macular edema is characterized by IRF retention in well-defined regions. Cystoid gaps are formed in the outer plexiform layer,^[31]^ whilst BALAD is located in the IS myoid layer.

However, the mechanism underlying BALAD formation remains unclear. Jung et al reported 2 main factors required to form a BALAD, including the potential space between the photoreceptor IS myoid bounded by the ELM and EZ and the hydrostatic force resulting from the rapid influx of exudative fluid from the choroid, generating stress that led to the splitting of the photoreceptor layer.^[13]^

Based on their review and picture analysis, Ramtohul et al suggested several hypotheses^[26]^ regarding the development of BALAD. Exudative SRF is initially caused by the destruction of the RPE component of the outer blood–retinal barrier. The intrinsically fragile IS structure is then separated during the generation of acute exudates, whereas ELM remains intact. DME has also been associated with SRF resulting from RPE breakdown.^[32]^ The cohabitation of BALAD and SRF observed in our study verified their association and was consistent with this hypothesis. Choroidal thickening has also been shown to be substantially correlated with BALAD,^[26]^ which has been linked to multiple disease processes, including those of inflammatory, ischemic, compressive, and neovascular origin. Choroidal thickness was not measured in this study. However, existing studies have shown that diabetic patients with DME have thicker choroids than those without DME.^[33]^ Among eyes with DME, SRF eyes exhibited the thickest choroid.^[33,34]^

High BALAD internal reflectivity and a high rate of suspended hyper-reflective particles likely indicate photoreceptor debris and inflammatory products.^[26]^ These findings of hyper-reflective contents led to the hypothesis that the accumulation of said contents modulated the adhesion between photoreceptor outer segment and RPE, and induced BALAD formation in these areas when hydrostatic pressure was exerted by subretinal exudation.^[26]^ Comparable to the assumption made by Ramtohul et al, the BALAD cavity contained hyperreflective material in the eyes of our study participants.

In this study, the baseline BCVA was comparable in all 3 groups. However, eyes with SRF (with and without BALAD) tended to have poorer baseline BCVA than those without SRF. Eyes with SRF had a substantial increase in baseline CFT relative to eyes without SRF, while eyes with BALAD among eyes with SRF demonstrated a more significant increase in CFT. This could be explained by the simultaneous presence of SRF and BALAD, as observed in every eye with BALAD in the present study. In addition, the degradation of the EZ structure was more extensive in the SRF-only group and the BALAD group than in the no either group (*P* = .023), making it a potential OCT biomarker that correlates with poorer baseline BCVA.

Intravitreal injection of anti-VEGF therapy has emerged as a first-line treatment for DME, while steroids play a subsequent or alternative role in the management of frequently refractory or chronically persistent DME.^[35–38]^ In this study, the response of BALAD patients after anti-VEGF therapy was collected, and there was no significant difference in the visual outcomes of the eyes across all groups. However, eyes in the BALAD subgroup exhibited relatively better results, followed by the SRF-only group and the no either group. This outcome correlates with the results of Sophie et al’s study^[39]^ that DME eyes with the presence of submacular fluid at baseline predicted an improved visual result in ranibizumab-treated individuals. Compared to the eyes of the no either group, the eyes in the BALAD group and the eyes in the SRF-only group displayed a substantial decrease in CFT relative to the baseline after 3 intravitreal injections of anti-VEGF. Following treatment, all 10 patients had complete clearance of BALAD, demonstrating an impressive response to therapy. A decrease in the SRF was observed, which in turn led to a reduction in the CFT. BCVA demonstrated a modest improvement, but we hypothesized that the improvement in BCVA was largely due to a decline in SRF rather than the resolution of BALAD. In addition, the development of EZ/IZ disruption could be observed after the resolution of BALAD, which would theoretically result in a decline in vision. But we postulate that the improvement in visual acuity induced by regression of SRF markedly outweighs the decrease caused by BALAD and the formation of EZ/IZ disruption following BALAD resolution.Moreover, Amini et al documented progressive restoration of the EZ during long-term follow-up, which may also explain the improvement in visual function after BALAD remission.^[23]^

Another recent study by Yordi et al demonstrated that the BCVA of AMD eyes with BALAD did not show improvement despite resolution of BALAD at 4 weeks after injection. The lack of improvement in BCVA was believed to be a result of EZ attenuation, as seen in the BALAD cases.^[27]^ However, unlike the BALAD found in AMD eyes, ours is relatively smaller in size and occurs within the area of much larger SRFs. We also noticed EZ attenuation following the resolution of the BALAD. We therefore hypothesize that the enhancement of vision caused by SRF resolution far cancels out the decline in visual acuity driven by EZ attenuation. Although the baseline SRF of the BALAD group was significantly higher than that of the other 2 groups, which should theoretically result in a more favorable visual prognosis, the less-than-desirable outcomes shown in our study may be related to EZ disruption.

The limitations of this study include the small sample size collected over a span of 6 years at 2 tertiary centers, the retrospective nature, and the short follow-up period of our study. As discussed in Ramtohul et al’s study,^[26]^ spontaneous regeneration of photoreceptors may be possible, so the actual number of BALAD eyes could hypothetically be underestimated. Additionally, the relatively small size of BALAD in our study compared to that of other previously reported disorders, the presence of exudations, and the presence of SRF all impact the diagnosis of BALAD because of the difficulty in distinguishing between distinct layers. The inherent risks of biases observed in retrospective studies include selection biases (we only included eyes with 3 anti-VEGF injections) or misclassification bias (the proper identification of BALAD). Owing to the very limited number of BALAD eyes found in our study, the clinical outcomes of BALAD may need further evaluation in a larger scale study for a definite assessment of this novel OCT finding. While the BCVA after anti-VEGF treatment yielded less than favorable results, structural improvements, such as CFT, still exhibited significant improvement. Further research would be necessary since the mechanism of BALAD formation is still unclear. A larger patient population and longer follow-up period are required for future clinical investigations to assess the function of BALAD in the pathophysiology of DME and as a predictor of visual prognosis.

## 5. Conclusions

In this retrospective study with short follow-up period, we enrolled treatment-naive DME patients who received 3 monthly anti-VEGF injections and employed SD-OCT to determine the prevalence and response to anti-VEGF treatment in eyes with DME with or without BALAD. According to the results of our research, the presence of BALAD was invariably related to SRF, with destruction of the EZ structure proposed as a potential OCT biomarker that correlates with poorer baseline BCVA. In addition to a significant improvement in CFT and a decrease in SRF following anti-VEGF treatment, a trend towards an improvement in BCVA was observed in eyes with BALAD, regardless of the development of EZ/IZ disruption after BALAD resolution. We postulate that the improvement in visual acuity induced by SRF regression significantly outweighs the decrease caused by BALAD and the formation of EZ/IZ disruption following BALAD resolution. In general, though after treatment and resolution of DME, eyes with BALAD developed disruption of the EZ/IZ in the region corresponding to BALAD, this finding did not appear to influence functional outcome. This early investigation revealed that bacillary detachment did not confer a negative predictive influence or response to treatment since BALAD eyes also had the most decrease in CFT. Therefore, to conclude, the presence or occurrence of BALAD in DME eyes does not appear to confer a negative prognostic or predictive value on visual outcome or CFT.

## Acknowledgments

The authors thank the clinical staff at the Cathay General Hospital and Tri-Service General Hospital for their excellent work.

## Author contributions

**Conceptualization:** I-Chia Liang.

**Data curation:** Yun-Hsiang Chang, Hsin-Ching Shen, Shu-I Pao, I-Chia Liang.

**Formal analysis:** Yann-Guang Chen, I-Chia Liang.

**Investigation:** Yann-Guang Chen, I-Chia Liang.

**Methodology:** Yann-Guang Chen, I-Chia Liang.

**Validation:** Yun-Hsiang Chang, Hsin-Ching Shen, Shu-I Pao, Yu-Chih Hou.

**Writing – original draft:** Yann-Guang Chen.

**Writing – review & editing:** Yu-Chih Hou, I-Chia Liang.
